# Debulking Surgery Combined with Low-Dose Oral Prednisolone and Azathioprine for Intractable IgG4-Related Orbital Disease: A Case Report

**DOI:** 10.3390/medicina57050448

**Published:** 2021-05-04

**Authors:** Hua-Hsuan Kuo, Chen-Hung Chen, Shu-Ya Wu

**Affiliations:** 1 Department of Ophthalmology, Taipei Tzu Chi Hospital, Buddhist Tzu Chi Medical Foundation, New Taipei City 231, Taiwan; serenahsuan@gmail.com; 2 Division of Allergy, Immunology and Rheumatology, Taipei Tzu Chi Hospital, Buddhist Tzu Chi Medical Foundation, New Taipei City 231, Taiwan; kasper4730@hotmail.com; 3 School of Medicine, Tzu Chi University, Hua-Liang County 970, Taiwan

**Keywords:** IgG4-related orbital disease, debulking surgery, azathioprine, prednisolone

## Abstract

A case of intractable IgG4-related orbital disease (IgG4-ROD) was successfully treated by debulking surgery combined with low-dose prednisolone and azathioprine as maintenance therapy. A 64-year-old man visited our clinic with progressive bilateral upper eyelid swelling and right eye fullness of a year’s duration. He was previously treated with systemic corticosteroids for the IgG4-ROD and experienced a partial clinical response but relapsed upon prednisolone cessation. The patient underwent debulking surgery of the right lacrimal gland and right upper eyelid. His post-operative medication was oral prednisolone (5 mg) every other day and 50 mg azathioprine per day. The patient’s right eye remained asymptomatic during the 18 months of follow-up. Debulking surgery combined with low-dose prednisolone and azathioprine, as a maintenance therapy, is an effective and alternative treatment for the long-term control of intractable IgG4-ROD.

## 1. Introduction

IgG4-related disease (IgG4-RD) is an immune-mediated systemic fibroinflammatory disorder characterized by dense lymphoplasmacytic infiltration with a predominance of IgG4-positive plasma cells, storiform fibrosis, and obliterative phlebitis [1]. IgG4-RD is usually a systemic condition, with frequent occurrence of orbital involvement [2,3]. IgG4-related orbital disease (IgG4-ROD) is most commonly reported in the lacrimal gland and in the extraocular muscles, trigeminal nerve branch, orbital soft tissue, eyelids, and nasolacrimal duct [4]. Corticosteroids are the preferred first-line systemic treatment for IgG4-ROD; however, up to 50% of cases experience symptom recurrence during the follow-up period [3]. Since IgG4-ROD is considered a systemic disease, the efficacy of debulking surgery is currently not fully understood. We report a case of IgG4-ROD involving the eyelid, supraorbital nerve, and lacrimal gland with an incomplete response to corticosteroid but a successful treatment with debulking surgery combined with low-dose prednisolone and azathioprine. 

## 2. Case Report

A 64-year-old man with a history of diabetes mellitus and glaucoma under oral Tristar and topical Xalatan and Azagar control presented with bilateral upper eyelid swelling and progressive right eye fullness for one year. The patient previously experienced a partial clinical response with oral prednisolone of 6 months’ duration for IgG4-ROD but relapsed upon prednisolone cessation. The oral prednisolone treatment also worsened his blood sugar control due to glucocorticoid-induced diabetes mellitus. He sought surgical intervention at our clinic after his symptoms worsened and after discontinuation of prednisolone.

**Figure 1:**
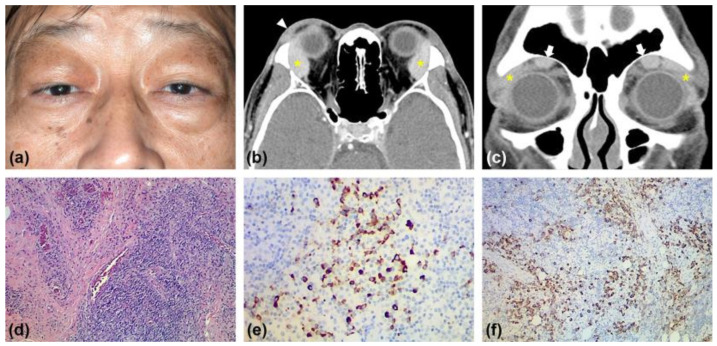
(**a**) Clinical appearance before surgery with bilateral upper eyelid swelling and a palpable mass over the right lacrimal fossa. (**b**) Contrast-enhanced axial computed tomography (CT) of the orbits revealed bilateral enlargement of the lacrimal gland (asterisk) and right upper eyelid’s homogenous enhancement with an irregular border (arrowhead). (**c**) Contrast-enhanced coronal CT of the orbits revealed bilateral enlargement of the lacrimal gland (asterisk) and supraorbital nerve (arrows). (**d**) Hematoxylin-eosin (H&E) staining demonstrated storiform fibrosis with diffuse lymphoplasmacytic infiltrate. (**e**) IgG4 immunohistochemical staining revealed >50 IgG4+ plasma cells /high power field and IgG4+/IgG+ ratio of >40% in the plasma cells infiltration. (**f**) Plasma cell immunohistochemical staining was positive for CD 138.

Regarding presentation, bilateral upper eyelid swelling and a palpable mass over the right sub-brow area were observed (Figure 1a). His best-corrected visual acuity was 20/63 bilaterally. The intraocular pressure measurement was 28 mmHg in the right eye and 20 mmHg in the left eye. The slit-lamp examination revealed the shallow anterior chamber with laser iridotomy and nuclear sclerosis of cataracts. He had no limited ocular motility. A contrast-enhanced computed tomography (CT) of the orbits revealed bilateral enlargement of the lacrimal gland and supraorbital nerve and homogenous enhancement with an irregular border of the right upper eyelid (Figure 1b,c). Laboratory results showed IgG4 level, 1970 mg/dL (3 to 201 mg/dL); IgG, 1980 mg/dL (700–1600 mg/dL); IgE, 65.9 IU/mL (<2–200 IU/mL); eosinophil, 7.8% (1–6%); and basophil, 1.3% (0–1%). Whole-body CT was negative (no retroperitoneal fibrosis, no lymphadenopathy, no submandibular and parotid gland enlargement, among others) except for the lesions in both orbits. Subsequently, the removal of the orbital lobe of the right lacrimal gland was performed via orbitotomy with a lateral canthal approach. Histopathology revealed storiform fibrosis with diffuse lymphoplasmacytic infiltrate (Figure 1d). IgG4 immunohistochemical staining revealed dense lymphoplasmacytic infiltrate with IgG4+ plasma cells > 50 cells/ high power field (HPF) and IgG4+/IgG+ ratio of >40% (Figure 1e) and was positive for CD 138 (Figure 1f). The pathology reports confirmed the diagnosis of IgG4-related orbital disease. 

**Figure 2:**
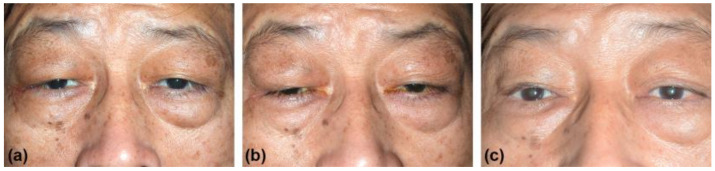
External photographs after orbitotomy (removal of the right lacrimal gland). (**a**) Three-week postoperative view showing reduced right upper eyelid swelling and proptosis. (**b**) Three-month postoperative view showing recurrent right upper eyelid swelling, and bilateral ptosis. (**c**) One-year postoperative view, six months after bilateral levator muscle advancement and right upper eyelid mass excision, showing marked improvement of right upper eyelid swelling and ptosis.

**Figure 3:**
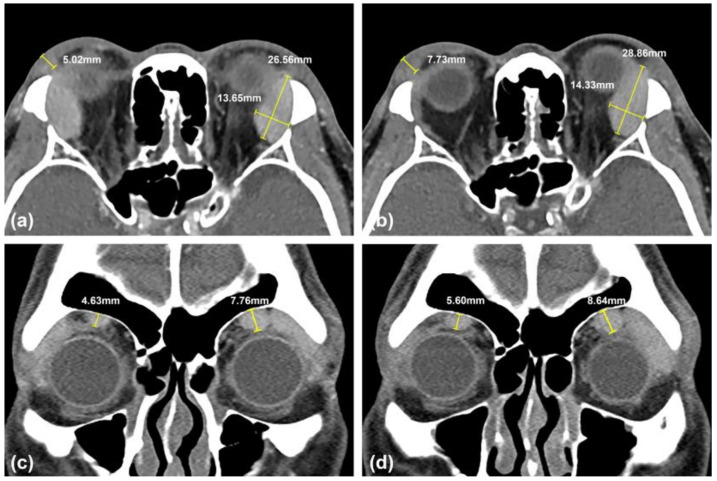
Contrast-enhanced CT of the orbits before orbitotomy (**a**,**c**) and six months after orbitotomy (**b**,**d**). (**a**) Axial view revealing bilateral enlargement of the lacrimal gland and right upper eyelid’s homogenous enhancement with an irregular border. The size of the left lacrimal gland was 26.56 mm × 13.65 mm. The thickness of the right upper eyelid mass was 5.02 mm. (**b**) Axial view revealing >90% reduction of the right lacrimal gland mass. The size of the left lacrimal gland was 28.86 mm × 14.33 mm. The thickness of the right upper eyelid mass was 7.73 mm. (**c**,**d**) Coronal view revealing bilateral enlargement of the supraorbital nerve. The diameter of the bilateral supraorbital nerve measured perpendicular to the frontal bone was 4.63 mm for the right eye and 7.76 mm for the left eye (**c**); 5.60 mm of the right and 8.64 mm of the left (**d**).

During the follow-up after surgery, the patient did not develop dry eye, and the mean intraocular pressure was 18 mmHg in the right eye and 21 mmHg in the left eye with the same medication as before surgery. Reduced right upper eyelid swelling and proptosis were observed three weeks after the surgery (Figure 2a). The patient was satisfied with the surgical result and resisted further immunosuppressive therapy. However, bilateral eyelid ptosis occurred three months after surgery (Figure 2b). A follow-up CT showed a >90% reduction of the right lacrimal gland mass but a slight increase in the thickness of the right upper eyelid, the size of the left lacrimal gland, and the diameter of the bilateral supraorbital nerve (Figure 3). The serum IgG4 level was 735 mg/dL. Due to the progressive enlargement of the tumor masses at a six-month interval and continued high levels of IgG4, oral prednisolone (5 mg) every other day and 50 mg azathioprine per day were administered. Two weeks later, the patient underwent simultaneous bilateral levator advancement and right tumor resection. The histopathological findings were consistent with IgG4-ROD of the right eyelid. After the second surgery, right upper eyelid swelling and bilateral ptosis showed a marked improvement (Figure 2c). The patient’s right eye remained asymptomatic and free of disease in the 18 months of follow-up.

## 3. Discussion

IgG4-ROD is a fibro-inflammatory condition, with tumefactive lesions, which mimics a variety of disorders. In particular, several studies have reported that the IgG4-ROD was associated with the extranodal marginal zone B-cell lymphoma [2,3]. It is essential to perform a biopsy surgery. Histopathology remains the gold standard for diagnosis. Treatment is often indicated to relieve the symptoms and to prevent complications. A diagnostic debulking surgery can provide sufficient tissues for histopathology and reduces the volume of tumor lesions to relieve the symptoms. In this case, IgG4-ROD was successfully treated by debulking surgery combined with a low dose of prednisolone and azathioprine as a maintenance therapy.

Currently, the first-line therapy for IgG4-ROD is systemic corticosteroid administration and the optimal initial dose of prednisolone is 0.6 mg/kg/day with a taper of 10% every two weeks [2,3,5]. The initial treatment response of corticosteroids is typically excellent; in the phase II clinical trial [5], 65.6% had complete remission and 27.6% had a partial response. However, relapses are common (26–50%) during the period of dose tapering or shortly after corticosteroid cessation [2,3]. Therefore, prednisolone at > 7 mg/day should be maintained [5]. As a result, 40% of patients experience newly diagnosed or worsening diabetes [5], as was observed for our patient. Rituximab is a monoclonal therapeutic antibody against CD20. It is usually initiated as a second- or third-line therapy for patients with refractive IgG4-ROD [3,5,6]. In the Wallace et al. [7] study, 95% of the patients had satisfactory clinical responses but 37% relapsed and required a repeat Rituximab treatment. Nevertheless, in our country, health insurance does not offer compensation for treatment. Other conventional disease-modifying anti-rheumatic drugs (DMARDs), including methotrexate, azathioprine, and mycophenolate mofetil, are generally used. Azathioprine is effective in some manifestations of IgG4-RD, such as pancreatitis, nephritis, cholangitis, and hypophysitis [8]. It is also the second most frequently used DMARD in IgG4-ROD’s treatment [3,6]. The treatment dose of azathioprine generally starts with 2.0–2.5 mg/kg/day for IgG4-RD [9]; however, its efficacy remains controversial with a reported response in the minority of patients with IgG4-ROD and a higher percentage of patients reporting side effects [3]. However, Park et al. [10] reported that prednisolone combined with an immunosuppressant such as azathioprine is effective for preventing recurrence. Recently, a meta-analysis study by Qmar et al. [11] also demonstrated that glucocorticoids combined with other immunosuppressive agents were associated with higher remission rates for IgG4-RD when compared with glucocorticoid alone.

Since IgG4-ROD is considered an immune-mediated systemic disease, the efficacy of debulking surgery is uncertain. Nevertheless, the tumor-like characteristics of IgG4-RD require surgery in certain cases where one solitary mass is observed, for example, a single pulmonary nodule [8]. In the systematic review study by Brito-Zeron et al. [12], most of 200 patients with surgery as a first-line therapeutic approach were diagnosed accidentally after removal of a potential tumor. The efficacy of surgery alone was 14/17 (82%). Ominato et al. [13], in 2019, also reported that debulking surgery might be an effective treatment for IgG4-dacryoadenitis with a relapse rate of 13.3%, which is much lower than the reported relapse rate (26–50%) of the prednisolone therapy. However, compared with the cases in the Ominato et al. [13] study, our case was more complicated with not only the lacrimal gland involvement but also involvement of the right eyelid and bilateral supraorbital nerves. Moreover, baseline elevations of the value of serum IgG4 (1970 mg/dL) and eosinophilia are strongly associated with the greater risk of subsequent relapse [7]. The follow-up orbital CT also demonstrated the slow enlargement of the orbital lesions without surgery. Studies revealed that surgery with immunosuppressive therapy had better odds of remission and less treatment failure than surgery alone [12,14]. Surgery was mainly an ancillary treatment for IgG4-RD. In our case, surgical debulking enabled resection of the mass lesion in the target organ and reduced the effects of local compression (reducing the intraocular pressure and fullness sensation). More than 90% of the orbital lobe of the right main lacrimal gland was resected without developing dry eye postoperatively. In general, it is believed that complete removal of the orbital lobe of the main lacrimal gland and leaving the palpebral lobe intact is insufficient to induce dry eye disease [15]. Low-dose prednisolone and azathioprine, as a maintenance therapy, was well tolerated by our patient with long-term use. Therefore, when Rituximab is not available due to economic restrictions, surgery combined with low-dose prednisolone and azathioprine, as a maintenance therapy, is an effective and alternative therapy for patients with diabetes mellitus who show an incomplete response and intolerance to glucocorticoids or who have a high risk of relapse.

## 4. Conclusions

In conclusion, IgG4-ROD is a systemic disease, and medical treatment with systemic glucocorticoids and immunosuppressants is the first treatment option to be considered. Debulking surgery combined with low-dose prednisolone and azathioprine may offer an alternative therapy for the patients with intractable IgG4-ROD who have an incomplete response or who relapse during prednisolone maintenance therapy.

## Data Availability

The datasets used during the current case report available from the corresponding author on reasonable request.

## Abbreviations

IgG4-ROD	IgG4-related orbital disease
IgG4-RD	IgG4-related disease
DMARDs	Disease-modifying anti-rheumatic drugs
HPF	High power field
CT	Computed tomography
